# Temporary nurse deployments: a time-series analysis of shift scheduling dynamics and staffing level alignment

**DOI:** 10.1016/j.ijnsa.2025.100383

**Published:** 2025-07-16

**Authors:** Maryam Ahmadi Shad, MohammadHossein Khorasanizadeh, Sarah N Musy, Franziska Zúñiga, Fatemeh Atoof, Michael Simon

**Affiliations:** a Institute of Nursing Science, Department of Public Health, University of Basel, Switzerland; b Trauma Nursing Research Center, Kashan University of Medical Sciences, Kashan, Iran; c Social Determinants of Health (SDH) Research Center, Kashan University of Medical Sciences, Kashan, Iran

**Keywords:** Nurse staffing, Temporary staff, Nursing management, Temporary nurses, Nursing workforce, Patient-to-nurse ratio, Time-series analysis

## Abstract

**Introduction:**

Hospitals deploy temporary nurses to bridge staffing gaps. However, evidence remains inconclusive regarding the extent, patterns, and factors driving temporary deployment. This study aimed to describe how temporary nurses are deployed as a response to shift-level schedule deviations and shortfalls in planned schedules.

**Methods:**

Our four-month time-series analysis covered 1344 shifts across two medical and two surgical units in a tertiary hospital in Iran. Shift-level data included nursing staff numbers, the skill mix, staff absences and the patient count and turnover. The patient-to-nurse ratio was used to gauge staffing levels. Data were analysed using both descriptive and analytical approaches, including the fitting of three generalized linear mixed models to assess potential drivers of shifts involving temporary RNs.

**Results:**

Temporary nurses worked on 12.2 % of shifts with the majority being Registered Nurses (RNs) (81.7 %). Only 28.5 % of deviations led to temporary RN deployments. While students and aides were sometimes reallocated to fill absences, the majority of absences (57.1 %) went unaddressed. Temporary staff mainly worked on shifts with below-average RN-staffing. Unit-level deployment rates varied widely (3.6 %–55.9 %). Model 1 revealed that RN absence increased the odds of using a temporary RN by 2.14 times (AIC = 785.1). Model 2 indicated that each additional patient, increased the odds by 11 % (AIC = 740.7). Model 3 showed that when RN-staffing was below-average the odds of using a temporary RN were 3.96 times higher than the average level (AIC = 707.4).

**Conclusion:**

Temporary nurse deployment was relatively infrequent. While temporary nurses were strategically deployed to address understaffing and short-notice deviations, their deployment did not fully bridge the staffing needs. On high-demand units, temporary staff were commonly supplemented by reallocating students. Some temporary deployments occurred even where RN-staffing was at an average level. These findings indicate an urgent need to enhance the effectiveness of temporary deployment and optimize workforce resources to ensure high-quality care.

What is already known

• The fluctuations in nurse staffing, which can be caused by peaks in demand as well as short-noticed absences, are important workforce issues in hospital setting.
• There is a rising trend of temporary nurse deployments in different hospital settings worldwide to fill staff vacancies.
• Deploying temporary staff is expensive and can affect patient safety outcomes.

What this paper adds

• The deployment of temporary nurses was relatively infrequent.
• This paper explores in detail the drivers of temporary nurse deployment using a data-driven approach. Temporary staff were used strategically to address both projected understaffing, and short-notice deviations from the staffing schedules.
• Deploying temporary staff only partially bridged the staffing gaps. In a significant majority of cases involving short-notice schedule deviations, no temporary staff was deployed. Surprisingly, though, some temporary deployments also occurred on shifts that already had average or above-average RN-staffing levels.
• The findings of this study highlight cases where staffing strategies that include the option of temporary staff deployment can be improved.

Alt-text: Unlabelled box

## Background

Hospital nursing staff accounts for nearly 50 % of the global health workforce (Boniol et al., 2022), making it the largest component of most healthcare teams. Particularly concerning patient safety and quality of care, it is crucial to ensure that each shift's nursing staff is adequate to meet patient needs (Dall’Ora et al., 2020). While a large body of literature has linked nurse staffing levels with patient outcomes, Dall’Ora et al.'s 2022 systematic review of longitudinal studies concluded that skill mix is also important: higher proportions of Registered Nurses (RNs) correlate with reduced risk of in-hospital mortality (Dall’Ora et al., 2022). Conversely, in an earlier review, Griffiths et al. (2016) found that inadequate levels of nurse staffing and poor skill mix correlated with adverse events attributed to nursing care. These included falls, longer lengths of stay, nosocomial infections, missed care, pressure ulcers, failures to rescue and drug administration errors (Griffiths et al., 2016).

N*ursing staff* refers to both the number and the skill mix of nurses (the supply). Each unit's overall daily supply includes all nursing staff on hand for that day's shifts, including RNs, nursing aides and students based on planned schedules (Dall’Ora et al., 2022). Nursing staff is scheduled in relation not only to the expected number of patients on the unit and their overall care demand (Griffiths et al., 2014), i.e., their combined clinical needs, but also to patient turnover (Musy et al., 2020). Because the numbers of patients admitted, discharged or transferred to and from a unit also contribute considerably to care demand.

Identifying effective and efficient strategies to balance the supply with demand is a top priority for all healthcare providers. However, unforeseeable variations—nurse absences, increases in demand, or combinations of the two—can still result in staffing shortfalls (Griffiths et al., 2021; Becker et al., 2010). When such gaps occur, hospitals can bridge them by calling in *temporary nurses*. These can be drawn from internal hospital pools, from permanently employed nurses who float between units, or from external agencies. Temporary nurses are deployed to maintain planned staffing levels when scheduled staff are unavailable or when supply/demand ratios fall below normal thresholds (Bae et al., 2015; Dall’Ora et al., 2020).

Current evidence regarding the extent, patterns and drivers of temporary nurse deployment is inconclusive. Because temporary staff use depends on factors such as nurse staffing management and operational demands, its prevalence differs widely across healthcare settings, units, hospitals and countries (Bae et al., 2015; Bowblis et al., 2024; Dall’Ora et al., 2020; Oliveira et al., 2023). For example, 25 % of units in Swiss psychiatric hospitals often deploy temporary nurses (Oliveira et al., 2023), compared with 76 % in medical/surgical units in the UK (Dall’Ora et al., 2020), or 41 % in ICUs in the US (Bae et al., 2015). This heterogeneity highlights the complex and context-dependent nature of temporary staffing practices. To our knowledge, though, no studies have yet investigated staffing levels and temporary nurse deployments in Iran.

Like many other countries, Iran faces considerable challenges with healthcare staffing in general, but particularly with nursing. Combined with uneven distribution of human resources across hospitals, supply shortages can reduce the quality of patient care (Nobakht et al., 2018; Zarea et al., 2009).

Despite research investigating the frequency of using temporary nurses and assessing temporary deployments' impacts on patient outcomes, few studies have explored when or how temporary nurses are assigned in practice. By delving into the dynamics of temporary nurse deployments, the current study goes beyond the reported rates of temporary staff use. It explores how they are deployed given the underlying staffing situations. Understanding the drivers which lead organizations to deploy temporary staff is crucial. For instance, which factor plays a more significant role: surges in demand or decreases in the available supply? Moreover, it is important to understand whether the deployment of temporary nurses is primarily driven by inherent understaffing—due to factors such as long-term vacancies, staff shortages, or a chronically high patient load—or by short-notice absences, which are more transient in nature. Distinguishing between these drivers enables hospital management to better identify the root causes of staffing gaps and implement targeted strategies to address them. This, in turn, can lead to improvements in both the quality of care and operational efficiency. Answering these questions will require first exploring how staffing schedules are planned, then learning how these schedules are implemented over time. Knowledge of the related processes will help to identify shift-level staffing gaps, as well as to determine how well strategic deployment of temporary nurses actually adjusts for staffing shortfalls.

This study's first aim was to better understand and describe the extent of temporary deployment in the studied units. The second aim was to explore two possible drivers of temporary deployment, i.e., to describe a) whether shift schedules were implemented as planned and, if not, to determine whether temporary staff were deployed as a response to deviations of the planned schedule; and b) to learn how temporary nurses were deployed across different staffing levels, and to determine whether it was a response to below-average staffing levels.

## Methods

### Study design and setting

This time-series analysis was conducted in a 650-bed tertiary hospital in Iran from April to July 2022. It is part of the ongoing TAILR (Nursing-Sensitive Events and Their Association With Individual Nurse Staffing Levels) project, an international research initiative to explore potential relationship between nurse staffing levels and nursing-sensitive adverse events (Bachnick et al., 2024). The current study included two internal medicine units (Neurology, Cardiovascular care) and two surgical units (General Surgery, Neurosurgery), each with at least 20 beds. Units providing higher levels of care (e.g., step-down, intermediate, and intensive care units) or offering highly specialized services (e.g., Oncology and Haematology) were excluded.

### Sample

Our analyses were based on routine shift-level staffing data collected over a four-month period, covering 336 shifts per unit. In the study hospital, all units follow a consistent three-shift model: morning shifts run from 07:15 to 14:15, afternoon shifts from 13:15 to 20:15, and night shifts from 19:15 to 08:15. According to Iranian working patterns in the hospitals, only Friday is considered a weekend day.

### Variables & measurement

Staffing data were extracted from each unit's paper-based scheduling documents. Two domains were analysed: supply and demand.

#### Supply domain

The supply domain encompassed the number of nursing staff (permanent or temporary) of all educational levels working in direct patient care, as well as any RN students doing internships on the study units. Nursing staff who worked less than a full shift (e.g., due to breastfeeding) were considered present if they completed more than half of the shift. In addition, the number of absences—including both last-minute call-outs and anticipated short-term absences—were recorded. Based on the available data it was not possible to define a threshold on when the absence was recorded and to clarify whether this was short-noticed.

Permanent nursing staff were categorized into three groups: 1) Registered nurses (RNs), 2) RNs with additional academic training (RN+) and, 3) Nursing aides. Temporary nursing staff were categorised into two groups: 1) Temporary registered nurses (RN or RN+) and, 2) Temporary nursing aides.

The study hospital's strategy to deploy temporary nursing staff is to float permanent staff between units to meet temporary increases in demand.

#### Demand domain

The demand domain refers to the number of patients hospitalized on the study units over the study period. All patients hospitalized over the observation period were included, regardless of their age, diagnosis or length of hospital stay. See Table 1 (below) for a full list of study variables.

**Table 1:** Description of variables contributing to nurse staffing.

Level	Variable	Definition
Supply		
	Permanent RNs	RN is defined as certified nurses who have completed a four-year university-based bachelor's degree in nursing.
	Absent permanent RNs	
	Temporary RNs	
	Permanent RNs+	RN+ is defined as certified nurses who have completed additional trainings including a master's degree in nursing.
	Absent permanent RNs+	
	Temporary RNs+	
	Permanent aides	Aide is defined as unlicensed personnel who have six to twelve months of vocational training, working under RN supervision.
	Absent permanent aides	
	Temporary aides	
	RN students	Bachelor students in their final year of education.
Demand	Patient number	Patients present in the unit at the beginning of the shift.
	Patient discharges	Patient discharges to out of the hospital setting.
	Patient admissions	Patient admissions from out of the hospital.
	Patient transfers in	Transfers into the study units from other units within the same hospital.
	Patient transfers out	Transfers from the study units to other units within the same hospital.

### Data collection

The staffing data collected for the four-month observation period was retrospectively extracted, using paper and pen, by the second author from each unit's scheduling documents for every day (from Saturday to Friday) and for every shift (morning, afternoon and night). Staffing data are routinely entered by the nurse in charge at the end of each shift.

To ensure accurate interpretation of the scheduling data, the research team discussed and verified them, as necessary, with the study unit managers. The second author then manually entered the data into this study's web-based electronic data capture platform. During the data cleaning phase, to ensure that all values were accurate and valid, the first author checked the dataset's plausibility regularly.

### Statistical analysis

#### Extent of temporary deployment

To fulfil our primary aim—to better understand and describe the shift-level extent of temporary deployment in the studied units—we calculated the frequency and percentage of shifts that included temporary RNs and aides per unit, day and shift.

#### Drivers of temporary deployment

To fulfil our second aim— to better understand and describe the conditions under which temporary staff are deployed in the studied units— specifically part (a), deviations from planned schedules were defined and assessed by calculating the frequency and percentage of shifts with short-notice absences. Absence data were broken down across shifts and days (weekdays vs. weekends), and were further categorized by staff qualifications (RNs, Aides) within each unit. The results are presented as bar charts, with percentages and frequencies of responses to absences categorized as “deploying temporary staff (RN(s) and/or aide(s)),” “increasing the number of students” or “no response.”

To address part (b) of our second aim— how temporary nurses were deployed across different staffing levels — we first empirically determined each unit's planned staffing levels, considering only shifts that occurred as planned, with no absents (1′227 shifts). We calculated patient-to-RN and patient-to-aide ratios by dividing the number of patients at the start of each shift by the number of nurses and aides available. We used the median and interquartile ranges (IQRs) to describe staffing variability (Musy et al., 2020) for each unit, shift, and weekday/weekend. Using the IQRs of these regular shifts as the target, we classified all shifts: shifts in the first quartile (Q1) were marked as “above-average staffing level” and with ratios in the third quartile (Q3) as “below-average staffing level”. As no policy-level or organizational standard for staffing levels was available for the target units, we categorized the shifts that fell within the IQR as staffed at an “average staffing level”.

We fitted three generalized linear mixed models to assess potential drivers of shifts with temporary RNs. All models included shift type as fixed effect and a random effect for the unit. Model 1 included whether RNs were absent on the supply side, model 2 for the number of patients as an indicator of the demand side and finally model 3 including the RN-staffing levels using within, above and below target categories as described above. As a sensitivity analyses, we fitted the same models and visualisation to a subset of the data which just included the shifts without any absences (1′227 shifts).

We conducted our data analyses using R software, version 4.1.1 for Windows. The following R packages were employed: dplyr (Wickham et al., 2022), tidyr (“Wickham and Girlich, 2022) and reshape2 (Wickham, 2025) for data preparation, and ggplot2 (Wickham, 2016), tableone (Yoshida and Bartel, 2022, 2022), and kableExtra (Zhu, 2021) for summarizing, tabulating and plotting the data. For the modeling analyses, the lme4 (Bates et al., 2015), rptR (Stoffel et al., 2017), and sjPlot (Lüdecke, 2021) packages were used to perform linear mixed-effects modeling, repeatability estimation, and data visualization, respectively.

## Results

### Deployment of temporary nurses

Over the four-month study period, our data encompassed a sample of 1344 shifts across four units.

Temporary staff (RNs or aides) were deployed on 164 shifts (12.2 %), with one to two temporary staff deployed for each affected shift. In the majority of shifts with temporary staff (81 %, 134 shifts), temporary RNs were deployed. Of the studied units, Unit 1 had the highest percentage of shifts (21.7 %) that used temporary staff. Compared to nights, temporary staff were deployed more frequently on morning (15.2 %) and afternoon shifts (12.5 %). There were no obvious differences in the deployment of temporary staff between weekdays and weekends. More details regarding temporary staff deployment are presented in Table 2. Fig. A (Appendix) shows how temporary staff deployments varied across the study period.

**Table 2:** Frequency of shifts with temporary staff across units, shifts and weekdays/weekends.

		Shifts with temporary staff		
		RN/RN+	Aide	Total*
Unit-level				
	Unit 1 (336)	67 (20 %)	11 (3.3 %)	73 (21.7 %)
	Unit 2 (336)	8 (2.4 %)	13 (3.9 %)	20 (6.0 %)
	Unit 3 (336)	9 (2.7 %)	3 (0.9 %)	12 (3.6 %)
	Unit 4 (336)	50 (14.9 %)	3 (0.9 %)	53 (15.8 %)
Day-level				
	Weekdays (1152)	115 (10 %)	25 (2.2 %)	137(11.9 %)
	Weekends (192)	19 (9.9 %)	5 (2.6 %)	21 (10.9 %)
Shift-level				
	Morning (448)	58 (12.9 %)	12 (2.7 %)	68 (15.2 %)
	Afternoon (448)	47 (10.4 %)	12 (2.7 %)	56 (12.5 %)
	Night (448)	29 (6.4 %)	6 (1.3 %)	34 (7.6 %)
	Overall	134 (10 %)	30 (2.2 %)	

*Number of shifts with at least one temporary staff (RN/RN+ or aide); The total does not match the sum of the two individual columns because, in some shifts, both a temporary aide and an RN/RN+ were present.

### Deviations from the planned shift schedules

Of the 1344 shifts analysed, 117 (8.7 %) deviated from the planned schedules. Of these, 96 (82.0 %) involved the absence of at least one RN. For nineteen shifts (16.2 %), one or more aides were absent, while in only 2 shifts (1.7 %), specifically in Unit 1, both an RN and an aide were absent. The frequency of shifts with RN absences per unit ranged from 3.6 % to 14.3 %, with Unit 1 accounting for the highest figures. Of the three shifts, mornings deviated most from the schedules (8.5 %), followed by afternoons (6.9 %) and, nights (6.5 %) (Table 3). Further details regarding aide absences are presented in Table A (Appendix).

**Table 3:** Frequency of shifts deviating from the planned RN/RN+ staffing schedules *(total shifts 1344 which corresponds to 336 shifts per unit)*.

	Shifts with absent RN/RN+			
	Morning (*n* = 448)	Afternoon (*n* = 448)	Night (*n* = 448)	All shifts with deviations*
Unit 1(Surgical)	19 (4.2 %)	17 (3.8 %)	12 (2.7 %)	48 (14.3 %)
Unit 2(Surgical)	4 (0.9 %)	6 (1.3 %)	8 (1.8 %)	18 (5.5 %)
Unit 3(Internal Medicine)	8 (1.8 %)	5 (1.1 %)	7 (1.6 %)	20 (5.9 %)
Unit 4(Internal Medicine)	7 (1.6 %)	3 (0.7 %)	2 (0.4 %)	12 (3.6 %)

*Total number of shifts with at least one RN/RN+ absent.

### Deviations from shift schedules and deployment of temporary nurses

**Fig. 1:**
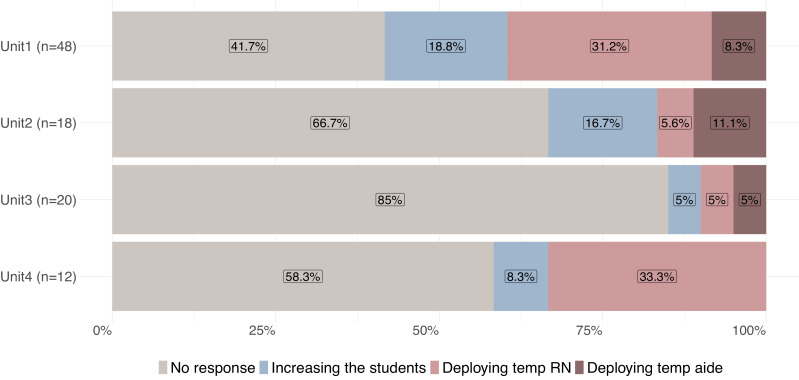
Managers’ responses to deviations in RN/RN+ staffing schedules.

Although RNs were absent from 98 shifts, over half of their absences (57.1 %) were not addressed, i.e., the most common response to a staffing gap was no response. The second most frequent response, which occurred on 21 shifts (21.4 %), was to deploy temporary RNs. In 14.3 % of cases of RN absences, the number of students was increased. And as the least frequent response (7.1 %), temporary aides were deployed. Fig. 1 shows the managers’ responses to deviations from the staffing schedules. Overall, temporary staff (RNs and aides) were deployed in only 28.5 % of shifts for which one or more RNs were absent. However, across the studied units, temporary RN deployment varied between 10 % in unit 3 and roughly 40 % in Unit 1. Of the 21 shifts with aide absences, the managers brought in extra students for only four (19.0 %). However, for the majority (81.0 %) of shifts, no response was made, i.e., no temporary staff, aides or students were supplied to any of the short-handed units (Fig. B, appendix).

Fig. A (Appendix) presents the numbers of students and temporary staff on the shifts with absences over the study period.

### Deployment of temporary staff in below-average, average and above-average RN-staffed shifts

We analysed a total of 1344 shifts. Table B (Appendix) presents the quartile-based threshold values, i.e., below-average, average and above-average RN-staffing levels, for different shifts on each unit across weekdays and weekends. Staffing patterns varied considerably across the units and shifts. On weekdays, below-average RN-staffing was more frequent in morning shift for Unit 4, afternoon shift for Unit 1, and night shift for Unit 3. In contrast, Unit 2 experienced a similar frequency of below-average RN-staffing across all shifts.

Over the weekend, Units 1 and 3 faced a similar frequency of below-average RN-staffing across shifts. However, Unit 4 showed improvement, with fewer below-average RN-staffing situations in the morning compared to the evening and night. Meanwhile, Unit 2 continued to face more staffing challenges during the weekend night shifts.

**Fig. 2:**
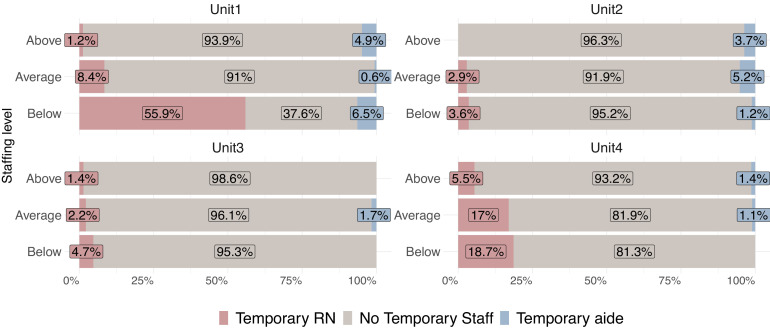
Frequency of temporary staff deployment across shifts with below-average, average and above-average RN–staffing.

When we examined the deployment of temporary nurses in relation to RN-staffing levels, we found that the highest proportion of temporary RNs (range: 3.6 % – 55.9 %) were deployed on below-average-staffed shifts (Fig. 2). As the results of a sensitivity analyses, Fig. C in the appendix shows the same graph with 1277 shifts (without absents).

According to the results of generalized linear mixed models analysis, in all three models we found significant relationships with temporary deployment (Table 4). In model 1, the results showed that when an RN is absent in the shift, the odds of using a temporary RN are 2.14 times higher compared to when there were no absent RN in the shifts (AIC=785.1). Model 2 shows that with an additional patient in the shift the odds of using–temporary RN increase by 11 % (AIC=740.7). In model 3 the results showed that when RN-staffing was above-average the odds of using a temporary RN were 79 % lower compared to when RN-staffing was average (AIC 707.4). On the other end, when RN-staffing was below-average the odds of using–temporary RN were 3.96 times higher than the average level. Additionally, all three models showed that night shifts have substantially lower likelihood of deploying temporary RN compared to morning shifts with odds of 0.46 %, 0.52 % and 0.39 %, respectively. Descriptive summary of the variables included in the model is presented in Table C in the appendix. Moreover, the sensitivity analyses showed very similar results presented in Table D in the appendix.

**Table 4:** Multilevel Logistic Regression to predict the drivers of temporary RN deployment.

Predictor	Model 1 OR (95 % CI)	p-value	Model 2 OR (95 % CI)	p-value	Model 3 OR (95 % CI)	p-value
Intercept	0.09 (0.02– 0.35)	<0.001	0.01 (0.00–0.03)	<0.001	0.07 (0.01–0.32)	<0.001
Shift with absent RN [Yes]	2.14 (1.19– 3.71)	0.008	—	—	—	—
Patient number	—	—	1.11 (1.07–1.14)	<0.001	—	—
RN-Staffing Level [Above]	—	—	—	—	0.21 (0.08–0.48)	0.001
RN-Staffing Level [Below]	—	—	—	—	3.96 (2.64–5.98)	<0.001
Shift [Evening]	0.79 (0.51–1.20)	0.270	0.95 (0.60– 1.47)	0.804	0.72 (0.46–1.13)	0.164
Shift [Night]	0.46 (0.27–0.73)	0.001	0.52 (0.31– 0.84)	0.009	0.39 (0.23–0.64)	<0.001
Random Effects						
τ00 Unit	1.05		1.63		1.23	
ICC	0.08		0.08		0.08	
Observations	1344		1344		1344	
Marginal R² / Conditional R²	0.033 / 0.268		0.114 / 0.407		0.181 / 0.404	

## Discussion

Temporary staff, including RNs and aides, were deployed on approximately one-eighth of the shifts in four study units over the four-month study period. When examining the conditions under which temporary staff were deployed, we investigated two perspectives: a) whether the deployments were in response to deviations from planned schedule (absences); or b) whether they were a response to below-average staffing levels.

We found that temporary staff were deployed in close to one-third of shifts that deviated from the RN-staffing plan due to absences, i.e., they addressed gaps resulting from absences. Furthermore, on all study units, the highest proportions of temporary RNs were deployed during shifts with below-average RN-staffing. This supports the explanation that temporary staff deployment was used as a strategy to address planned and short-noticed staffing deficiencies.

Our findings concerning temporary nurse deployments align generally with previously-identified temporary staffing trends in hospital settings; however, our overall prevalence differs notably from those noted elsewhere. For instance, while Aiken et al.'s cross-sectional study reported that up to 32.7 % of shifts involved temporary nurses (Aiken et al., 2007), Bae et al. demonstrated that 75 % of medical and surgical units relied on them (Bae et al., 2010). Similarly, Oliveira et al. highlighted the frequency of temporary nurse deployment: one-quarter of their study units regularly utilized temporary staff (Oliveira et al., 2023). Dall’ora et al. also reported a high reliance on temporary staff, with 76 % and 87 % of unit days including at least a half hour of patient care provided, respectively, by temporary RNs or nursing assistants (Dall’Ora et al., 2020). However, the literature presents a mixed picture. More consistent with our findings, Senek et al. showed that 71 % of shifts had no temporary agency staff, indicating a much lower reliance on temporary staff (Senek et al., 2020). Similarly, Hurst and Smith found that temporary staff accounted for 14 % of ward teams (Hurst and Smith, 2011); and more recently, Griffith et al. reported that temporary staff were used for approximately 10 % of all patient days, with nearly 14 % of nursing assistant hours provided by temporary staff (Griffiths et al., 2024).

However, these findings should be interpreted with caution: their diversity reflects the heterogeneity of the methodologies used to define and measure temporary deployments. Still, while the reported numbers appear comparable, their methodological differences prevent direct comparisons. Additionally, where the cited studies used cross-sectional designs, their methodological robustness is limited, precluding any inferences of causality. Of the longitudinal studies that report rates of temporary staff deployment, few address patterns of use or explore the drivers of using temporary nurses.

Another factor magnifying the differences between our findings and those reported by earlier researchers could be the Iranian health system's distinct concept of temporary staffing, which likely reduces the prevalence of temporary staff deployment there compared to those in other countries. In countries with well-established temporary staffing systems, external agencies or internal pools ensure temporary supply and manage temporary nurses deployment. We assume that such approaches contribute to higher and very likely more consistent use of temporary staff across healthcare settings.

More interestingly, our study also highlights the complexity of temporary staff deployment, as it varied significantly across different units and shifts even in the same hospital. One of the surgical units included in this study experienced a high rate of short-notice absences, particularly during morning shifts. This led to a higher frequency of shifts with below-average RN staffing levels, which in turn increased the unit’s reliance on temporary staff. Even though temporary nurses were deployed almost exclusively on morning and afternoon shifts, the demand often exceeded supply. As a result, many shifts remained understaffed. This shows that the hospital’s temporary staffing system was not able to handle the high variability in nurse staffing. For cases such as this, where short-term staffing gaps are unevenly distributed even across units in the same hospital, regular analysis of the relevant data will allow hospital managers to develop proactive strategies to bridge gaps in the supply, thereby safeguarding patient safety.

Much of the literature on temporary nurse deployments implies consistent reasons of use, e.g., to cope with hospital staffing deficiencies. However, Bajorek and Guest found that using temporary staff as a substitute for recruiting new staff can lead to chronic shortages of permanent staff. Additionally, their study hospitals employed temporary staff to cover for sick days, holidays, and other types of absences, or to soften the effects of hiring delays resulting from lengthy recruitment processes (Bajorek and Guest, 2019). Similarly, studies conducted in the UK and Switzerland have identified staff shortages caused by short-term absences (illness/accident), unfillable vacancies, and increased workload as the main drivers behind temporary staff deployments (Ahmadi Shad et al., 2023; National Audit Office, 2006). However, these findings are based primarily on surveys and conducted in contexts vastly different from those of Iranian hospitals. Likewise, the workforce management and staffing challenges faced in these countries very likely differ from those in Iran.

For this study, we explored two key scenarios. First, we considered deviations from staffing schedules in the form of short-notice absences or gaps that would commonly lead to temporary staff deployments. Second, we analysed cases where, even if no absences occurred, the planned staffing did not meet the demand. Specifically, we hoped to determine whether temporary staff deployment was applied as a strategy to overcome such situations.

We found that almost one-tenth of shifts involved deviations from the staffing schedule. Where unit managers addressed these deviations, they employed a variety of responses. In one-third of deviated shifts, unit managers bridged the gaps either by increasing the number of students on hand or by deploying temporary RNs. Otherwise—as occurred in nearly two-thirds of shifts with absent RNs—the shifts simply remained understaffed. Several factors could have contributed to leaving these shifts without substitution, including the extent of understaffing, the availability (or lack) of substitution —temporary staff or students—, the overall care demand load, and unit-specific management policies. In some cases, unit managers may have opted to continue operations with the remaining staff based on these considerations.

In comparison, when Senek et al. analysed survey data on 8841 shifts across the UK, they found that 61 % of those shifts diverged to some extent from the staffing schedule, with agency staff constituting between 0.3 and 0.5 proportion of the workforce, depending on the extent of understaffing (Senek et al., 2020). Similarly, Hurst and Smith found that, among the participating units that relied substantially on temporary staff, many also had notable (>20 %) absence rates among permanent staff (Hurst and Smith, 2011). Also, our findings suggest that our study units' managers employed various strategies to deploy temporary staff on shifts with RNs absent. As one surgical and one internal medicine unit had notably higher rates of temporary staff deployment, we suspect that these differences reflect unit-specific policies regarding staffing shortfall management.

In cases where RNs were absent but temporary staff were not deployed, unit managers had two alternatives: either they could attempt to manage the care demand using the remaining staff, or they could reallocate RN students from other units. Problematically, though, while nursing students in their final year have received extensive training, they cannot replace experienced professional staff. Attempting to do so raises concerns about patient safety and quality of care.

The high incidence of failure to fully address deviations from scheduled staffing highlights the complexity of managing replacements over short-term absences. This observation is not unique to Iran: preliminary findings from a sister study in Switzerland reveal a similar pattern.

To date, no consensus exists on the definitions of low, average or high staffing levels. Therefore, acknowledging that normal nurse staffing levels vary not only across units, but also between weekdays and weekends and between different shifts, we chose to define high and low patient-to-nurse ratios contextually, based on quartiles. A similar strategy was used by Musy et al. (2020) who observed comparable trends in Swiss hospitals (Musy et al., 2020). On some units, the instability observed in staff schedules corresponded to resource constraints; in others, deviations were more likely to reflect higher workloads on specific shifts or days.

Across all units, the highest proportions of temporary RNs were deployed during shifts with below-average RN-staffing, suggesting a strategic use of temporary RNs to fill scheduling gaps. Strategic deployment in this context means, that temporary nurses are deployed based on the targeted staffing level, which is not necessarily driven by actual demand (e.g. through high occupancy and/or case severity). However, we acknowledge that this term may imply a level of deliberate planning or policy-driven intent that cannot be fully confirmed from our data. As such, we use it to describe observable patterns of deployment rather than to infer formal organizational strategy.

Among the units, one surgical and one internal medicine unit demonstrated more effective strategic responses to variations in staffing. Compared to their counterparts with low RN-staffing levels, these units' shift-level data on temporary deployments reflected either more efficient management practices or better access to substitutes. It may also be possible that the other two units employed alternative strategies than temporary nurse deployments, such as staff overtime, to meet staffing needs. Supporting our findings, Aiken et al. found that hospitals with fewer resources, including lower staffing levels, tended to rely more on non-permanent nurses (Aiken et al., 2007). And Hurst and Smith highlighted a clear relationship between increased care demand and temporary staff deployment (Hurst and Smith, 2011). Conversely, Griffiths et al. found that while nearly 10 % of their sample's RN hours were provided by temporary staff overall, this figure was actually slightly (approximately 1 %) lower during days of low staffing status (Griffiths et al., 2024). Although they concluded that temporary staff can effectively fill some gaps in planned staffing levels, it is crucial to evaluate how large those gaps can be and over what periods such deployments can effectively make up for such shortfalls.

In almost all units, proportions of temporary RNs were deployed also during shifts with average or above-average RN-to-patient ratios, when staffing levels appeared adequate. This may be attributed to other factors of nursing workload not captured by our metrics, e.g. patient acuity.

## Strengths and limitations

This study is the first of its kind to explore the phenomenon of temporary staffing and its dynamics in Iran. On a broader scale, it attempts to describe patterns of responses to staffing gaps via temporary staffing deployment. While nurse floating is common in hospitals in Iran, the nurses floated between units are not typically classified as temporary staff. Our time-series analysis allowed us to investigate temporary deployments thoroughly in relation to fluctuations in staffing adequacy in relation to patient needs, i.e., imbalances in supply vs. demand.

One key limitation of this analysis was the lack of precise data regarding the hours worked by temporarily deployed nurses. Particularly for cases where temporary nurses were assigned to units for brief periods—often two hours or less—no data were available. Only those nurses who worked the entire shift were documented. This data gap may have led us to underestimate the rates of temporary deployments. Another potential area of interest that we were unable to explore was the question of whether the temporary nurses' specialised skills matched the requirements of the units to which they were assigned. Unfortunately, we did not have access to sufficient detail regarding the temporary staff to comment on that point.

In this study our primary aim was to gain a clearer understanding of the current situation of temporary nurse staffing practices. We did not incorporate nurse- or patient-related outcomes, which is a limitation. The impact of exposure to temporary nursing staff on the quality of care and patient safety outcomes will be explored in the next phase of this project.

## Conclusion

Our findings indicate relatively infrequent deployment of temporary nurses. Often, temporary staff were used strategically to address both projected understaffing, and short-notice deviations from the staffing schedules. The results of our data analyses suggest that temporary staff only partially bridged staffing gaps. However, in a significant majority of cases involving short-notice schedule deviations, staffing gaps were simply left open. In some cases of RN absences on high-demand units, their managers attempted to bridge the resulting gaps by re-allocating students from lower-demand units.

Furthermore, while our analyses suggested that temporary staff were often strategically deployed on shifts with below-average RN-staffing, a significant proportion of these shifts remained understaffed due to initial planning limitations. Surprisingly, though, some temporary deployments also occurred on shifts that already had average or above-average RN-staffing levels. These findings highlight cases where staffing strategies that include the option of temporary staff deployment can be improved. To ensure continuous, high-quality patient care, it is crucial to develop and implement robust, proactive staffing solutions that can better address care gaps and optimize workforce resources.

### Implications for management and research

Our findings indicate that although temporary nurse deployments were strategically applied, they did not fully bridge the staffing gaps.For managers to optimise their resource use, rather than relying on less qualified replacements, such as students, or simply leaving shifts understaffed, we recommend that managers adopt more strategic approaches to temporary staff use. At the departmental or institutional level, it is crucial to establish clear staffing policies that allow units incorporate flexible staffing strategies to address fluctuations in staffing levels. This, however, requires stronger support and training for nursing managers, particularly within the Iranian healthcare context.

In Iran, despite holding managerial responsibilities, nursing managers are often selected based on clinical experience rather than leadership or administrative qualifications. Moreover, they are expected to spend plenty of their time on clinical duties as well, which limits their capacity to focus on administrative and strategic planning tasks. Addressing this imbalance through improved training, structured support systems, and succession planning could significantly enhance their effectiveness (Barasteh et al., 2021).

Especially regarding temporary staffing, Iran may lack the formalised mechanisms available in other healthcare systems, such as internal hospital staffing pools or structured processes for temporary staff deployment. Still, well-defined policies would enable its hospitals to respond more effectively both to workforce shortages and varying demand across units.

At the macro level, addressing the root causes of staffing challenges—the nursing shortage—requires systemic reform. Key strategies should include increasing the enrolment of nursing students, revising employment policies, decentralizing the health system, and improving nurse compensation. These measures, as highlighted in the current researches, are essential for building a more resilient and responsive nursing workforce in Iran (Abbaszadeh and Abdi, 2017; Shamsi and Peyravi, 2020).

Future research should investigate how temporary staffing practices vary across different healthcare settings on national and international levels. While our findings suggest that temporary staff were used mainly to understaffed situations and absences, further research will be necessary to assess whether these temporary deployments can fully contribute to address such shortfalls. Especially in terms of team functionality, workload balance and patient safety outcomes, it is important for future research to analyse whether the care provided by temporary nurses is comparable to that provided by permanent staff. This represents a valuable avenue for further investigation in the field.

## Ethical approval

Ethical approval for the study was obtained from the Ethics Committee of Kashan University of Medical Sciences (TAILR.IR: IR.KAUMS.REC.1398.032). The research team provided the study hospital with the written permissions acquired from the Ethics Committee for collecting the data.

## CRediT authorship contribution statement

**Maryam Ahmadi Shad:** Writing – review & editing, Writing – original draft, Visualization, Validation, Supervision, Project administration, Methodology, Formal analysis, Data curation, Conceptualization. **MohammadHossein Khorasanizadeh:** Writing – review & editing, Validation, Resources, Project administration, Investigation, Conceptualization. **Sarah N Musy:** Writing – review & editing, Visualization, Validation, Software, Methodology, Formal analysis, Data curation, Conceptualization. **Franziska Zúñiga:** Writing – review & editing, Validation, Supervision, Methodology, Conceptualization. **Fatemeh Atoof:** Writing – review & editing, Validation, Supervision, Project administration, Funding acquisition, Conceptualization. **Michael Simon:** Writing – review & editing, Validation, Supervision, Project administration, Methodology, Funding acquisition, Data curation, Conceptualization.

## Declaration of competing interest

The authors declare that they have no known competing financial interests or personal relationships that could have appeared to influence the work reported in this paper.

## Data Availability

Due to the sensitive nature of the data which is analyzed in the current study, and the lack of permission from the ethics committee of the study site to share it publicly, the data is not available.

## Appendix Supplementary materials

Image, application 1
