# Supplementary material for: Temporary nurse deployments: a time-series analysis of shift scheduling dynamics and staffing level alignment
Source: Int J Nurs Stud Adv. 2025 Jul 16;9:100383. doi: 10.1016/j.ijnsa.2025.100383 (PMC12410400; doi:10.1016/j.ijnsa.2025.100383)
Supplement: Supplementary file 1 [file mmc1.docx]

# Appendix. Supplementary materials

Table A: Frequency of the shifts deviating from the aide-staffing schedules

| Unit | Shifts with absent aides | | | | | |  |
| --- | --- | --- | --- | --- | --- | --- | --- |
|  | Morning  (n = 448) | | Afternoon  (n = 448) | | Night  (n = 448) | | All shifts with deviations* |
|  |  |  |  |  |  |  |  |
| Unit1 | 1 (0.2%) | | 1 (0.2%) | | 2 (0.4%) | | 4 (1.2%) |
| Unit2 | 5 (1.1%) | | 5 (1.1%) | | 4 (0.9%) | | 14 (4.2%) |
| Unit3 | 0 (0.0%) | | 0 (0.0%) | | 0 (0.0%) | | 0 (0.0%) |
| Unit4 | 3 (0.7%) | | 0 (0.0%) | | 0 (0.0%) | | 3 (0.9%) |

*Total number of shifts with at least one aide absent

Table B: Comparison of the shifts with below average, average and above average RN-staffing level across units, shifts and weekdays/weekends

|  |  |  | Median (IQR) of patient/RN ratios |  |  |  |
| --- | --- | --- | --- | --- | --- | --- |
| Unit |  | Shift |  | Above average | Average | Below average |
| Unit1  (Surgery) | Weekdays | Morning | 3.5 (2.7-4.8) | 26 (27.1%) | 49 (51.0%) | 24 (25.0%) |
|  |  | Afternoon | 4.8 (3.7-7.1) | 22 (22.9%) | 46 (47.9%) | 28 (29.2%) |
|  |  | Night | 4.7 (4.0-6.6) | 22(22.9%) | 48 (50.0%) | 26 (27.1%) |
|  | Weekends | Morning | 4.5 (3.6-6.6) | 4 (25.0%) | 8 (50.0%) | 4 (25.0%) |
|  |  | Afternoon | 5.7 (4.3-7.6) | 4 (25.0%) | 8 (50.0%) | 4 (25.0%) |
|  |  | Night | 4.3 (3.6-6.5) | 4 (25.0%) | 8 (50.0%) | 4 (25.0%) |
|  |  |  |  |  |  |  |
| Unit2  (Surgery) | Weekdays | Morning | 4.4 (3.6-4.8) | 23 (24.0%) | 49 (51.0%) | 24 (25.0%) |
|  |  | Afternoon | 6.7 (5.5-7.3) | 22 (22.9%) | 50 (52.1%) | 24 (25.0%) |
|  |  | Night | 6.7 (5.6-7.3) | 23 (24.0%) | 49 (51.0%) | 24 (25.0%) |
|  | Weekends | Morning | 6.0 (5.0-7.0) | 4 (25.0%) | 10 (62.5%) | 2 (12.5%) |
|  |  | Afternoon | 7.3 (5.8-8.0) | 5 (31.2%) | 8 (50.0%) | 3 (18.8%) |
|  |  | Night | 6.5 (5.4-7.5) | 4 (25.0%) | 6 (37.5%) | 6 (37.5%) |
|  |  |  |  |  |  |  |
| Unit3  (Internal Medicine) | Weekdays | Morning | 5.7 (4.8-7.0) | 17 (17.7%) | 57 (59.4%) | 22 (22.9%) |
|  |  | Afternoon | 8.3 (7.3-9.8) | 21 (21.9%) | 50 (52.1%) | 25 (26.0%) |
|  |  | Night | 8.6 (8.0-10.6) | 23 (24.0%) | 47 (49.0%) | 26 (27.1%) |
|  | Weekends | Morning | 8.6 (8.1-9.6) | 4 (25.0%) | 8 (50.0%) | 4 (25.0%) |
|  |  | Afternoon | 8.6 (7.6-9.9) | 3 (18.8%) | 9 (56.2%) | 4 (25.0%) |
|  |  | Night | 8.3 (7.3-10.3) | 3 (18.8%) | 9 (56.2%) | 4 (25.0%) |
|  |  |  |  |  |  |  |
| Unit4  (Internal Medicine) | Weekdays | Morning | 4.4 (3.8-5.0) | 16 (16.7%) | 57 (59.4%) | 23 (24.0%) |
|  |  | Afternoon | 6.0 (5.2-6.5) | 24 (25.0%) | 52 (54.2%) | 20 (20.8%) |
|  |  | Night | 6.2 (5.5-6.7) | 22 (22.9%) | 52 (54.2%) | 22 (22.9%) |
|  | Weekends | Morning | 6.5 (5.7-6.7) | 4 (25.0%) | 9 (56.2%) | 3 (18.5%) |
|  |  | Afternoon | 6.1 (5.3-7.0) | 4 (25.0%) | 8 (50.0%) | 4 (25.0%) |
|  |  | Night | 6.9 (5.7-7.5) | 3 (18.8%) | 10 (62.5%) | 3 (18.8%) |
|  | Overall |  |  | 307(22.8%) | 706 (52.5%) | 331 (24.6%) |

Table C: Description of the variables used in the models

|  | Variable | Mean (SD) | Median [Q1-Q3] | N (%) |
| --- | --- | --- | --- | --- |
| Outcome | Shift with temporary RN | - | - | No= 1210 (90.0%)  Yes= 134 (10.0%) |
|  | Shift | - | - | Morning:448 (33.0%)  Evening:448 (33.0%)  Night:448 (33.0%) |
|  | Shifts with RN absent | - | - | No= 1246 (92.7%)  Yes= 98 (7.3%) |
|  | Number of patients | 23.15 (6.71) | 24.00 [19.00-27.00] | - |
|  | RN-staffing levels | - | - | Above-average :307 (22.8%)  Average:706 (52.5%)  Below-average :331 (24.6%) |

Table D: Multilevel Logistic Regression to predict the drivers of temporary RN deployment (only shifts without absents as a sensitivity analysis)

| Predictor | Model 1  OR (95% CI) | p-value | Model 2  OR (95% CI) | p-value | Model 3  OR (95% CI) | p-value |
| --- | --- | --- | --- | --- | --- | --- |
| Intercept | 0.10 (0.02– 0.39) | <0.001 | 0.00 (0.00–0.04) | <0.001 | 0.08 (0.01–0.34) | <0.001 |
| Shift with absent RN [Yes] | — |  | — | — | — | — |
| Patient number | — | — | 1.10 (1.07–1.14) | <0.001 | — | — |
| RN-Staffing Level [Above] | — | — | — | — | 0.20 (0.07–0.48) | 0.001 |
| RN-Staffing Level [Below] | — | — | — | — | 3.53 (2.28–5.48) | <0.001 |
| Shift [Evening] | 0.65 (0.40–1.03) | 0.067 | 0.79 (0.49– 1.29) | 0.361 | 0.61 (0.37–1.00) | 0.053 |
| Shift [Night] | 0.41 (0.24–0.67) | 0.001 | 0.48 (0.28– 0.82) | 0.008 | 0.37 (0.21–0.63) | <0.001 |
| Random Effects |  |  |  |  |  |  |
| τ00 Unit | 1.06 |  | 1.50 |  | 1.16 |  |
| ICC | 0.07 |  | 0.07 |  | 0.07 |  |
| Observations | 1227 |  | 1227 |  | 1227 |  |
| Marginal R² / Conditional R² | 0.030 / 0.266 |  | 0.105 / 0.386 |  | 0.196/ 0.406 |  |

Figure A: Numbers of student and temporary staff deployments during shifts deviating from staffing schedules.

Note: Vertical dotted line = Shifts with absent staff, Grey area = weekend (Friday), Black horizontal line = median number of students

Figure B: Managers’ Responses to deviations in aide-staffing schedules

Note: The median numbers of students per unit, month, and day were calculated to establish a baseline for normal student levels. The frequencies of shifts exceeding these median student counts were then described for shifts affected by absences.

Figure C: Frequency of temporary staff deployment across shifts with below-average, average and above-average RN-staffing (only shifts without absents as a sensitivity analysis)
